# News and Views

**DOI:** 10.1007/s43673-021-00008-1

**Published:** 2021-03-18

**Authors:** AAPPS

**Affiliations:** Association of Asia Pacific Physical Societies, Pohang, Korea

## ANPhA/AAPPS-DNP activities and some news from the Asia Pacific nuclear physics community

The Asian Nuclear Physics Association (ANPhA) was established in 2008. It represents nuclear physics in the Asia Pacific region. It also plays a role as the Division of Nuclear Physics (DNP) of AAPPS. Because of COVID-19 situations, its regular activities are limited. For example, the annual board meeting scheduled in December will be organized virtually by the University of Hong Kong (HKU). From the year 2020, Prof. Weiping Liu from China served as the ANPhA chair, succeeding Prof. Tanaka from Japan, who has made great contributions to the activities. Maintenance of the ANPhA website was moved from RIKEN in Japan to CIAE in China (https://bisol.org/anpha/index.html). The site has updated information supposed to be useful for nuclear physics researchers in the region, such as in terms of job opportunities.

Despite the difficult situation caused by COVID-19, ANPhA continued to spiritually support international conferences and symposiums held in the Asia Pacific: NIC2021 conference in Chengdu, CNS Summer School 2020 organized online by CNS, SNP school 2020 in Tokai (partially online), APLAT 2020 symposium organized online by KEK, NuSYS summer school 2021 in Beijing, and APFB2020 scheduled to be held in the city of Kanazawa in 2021. Thanks to the AAPPS supports, together with the budgets collected in previous years, ANPhA/DNP awards were granted to young scientists in Asia on the occasions of some of the ANPhA-supported symposiums.

A report on ANPhA activities for these 10 years was published in NuPECC’s Nuclear Physics News, entitled “Ten Years of the Asian Nuclear Physics Association (ANPhA) and Major Accelerator Facilities for Nuclear Physics in the Asia Pacific Region” [1]. The article was written by several board members and edited by the former Chair Prof. Tanaka. Another publication to review the progress of nuclear astrophysics in Asia is in preparation for the AAPPS Bulletin in its “Review and Research” section. Some members of the ANPhA board are also involved.

**Fig. 1:**
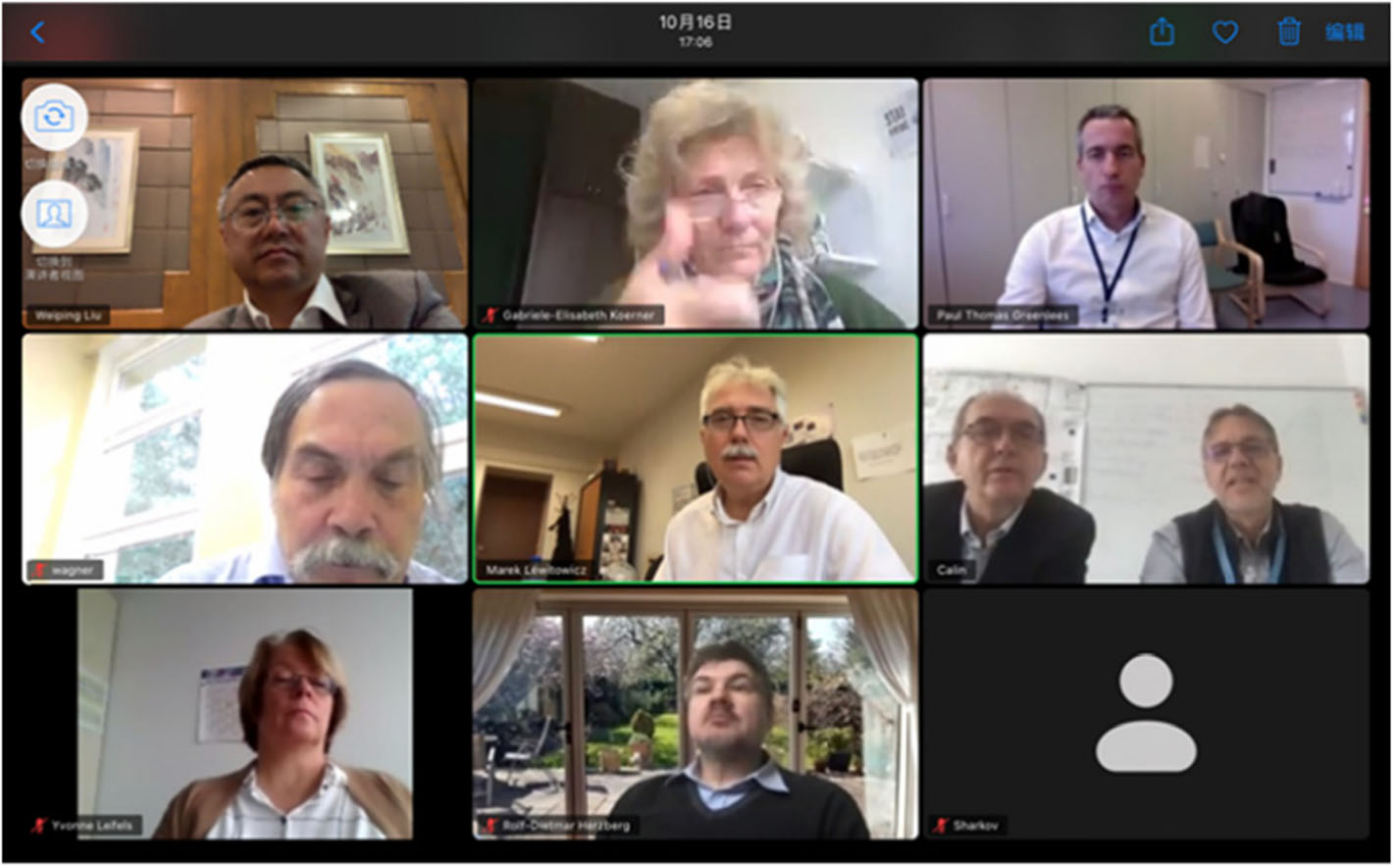
A snapshot of the NuPECC web meeting

As an observer from ANPhA, Prof. Weiping Liu participated in the Nuclear Physics European Collaboration Committee (NuPECC) board meeting held on October 16 (see Fig. 1). ANPhA and NuPECC regularly exchange observations in their the meeting.

Great news in 2020 for the nuclear physics community was the AAPPS Chen Ning Yang Award, which was received by Nobuyuki Kobayashi from RCNP Osaka for his outstanding contributions to exotic nuclear physics.

On December 1, Weiping Liu reported the ANPhA activities as a DNP division chair, in the AAPPS video council meeting.

Another news was for Asian-wide nuclear physics collaborations under the “A3 Foresight Program” that started in 2019. They are based on an agreement among the Japan Society of Promotion of Science (JSPS), the National Research Foundation of Korea (NRF), and the National Natural Science Foundation of China (NSFC). It supports the joint research conducted by researchers from Japan, China, and Korea. The program aims at establishing a top-level research hub in Asia (see https://www.jsps.go.jp/english/e-foresight/index.html). Two nuclear physics programs are adopted under the titles of “Reaction dynamics towards the limits of nuclear and elemental existences” and “Various Manifestations of Nuclear Structure -- From Nucleons to Nuclear Matter at in Extreme Conditions”.

## Young Scientist Award of the Physical Society of Japan, 2021

Every year, the Physical Society of Japan presents Young Scientist Award for young researchers who have made outstanding achievements in their early research careers. This year’s winners were recently decided by the board of directors of the JPS on the recommendations of the selection committees established in 19 divisions of the JPS. The maximum number of winners from each division has been determined on the basis of the number of talks delivered in the annual meetings of the past 3 years. All the winners will deliver an award lecture at the next annual meeting of the JPS scheduled for March 2021. Here is the list of winners and their research topics.

1. Theoretical particle physics:
  - Yohei Ema (The Deutsches Elektronen - Synchrotron), “Scalaron in Higgs Inflation”
  - Masatoshi Yamada (Heidelberg University), “Quantum Gravity Effect in a Scenario with Asymptotic Safety and its Application to an Extended Higgs Model”
  - Chang-Tse Hsieh (Kavli Institute for the Physics and Mathematics of the Universe/The Institute for Solid State Physics, The University of Tokyo),“Anomaly of the Electromagnetic Duality of Maxwell Theory”
2. Experimental particle physics:
  - Yosuke Ashida (Kyoto University), “Measurement of Neutrino and Antineutrino Neutral-Current Quasielastic-like Interactions and Applications to Supernova Relic Neutrino Searches”
  - Masahiko Saito (The University of Tokyo), “Search for direct Chargino Production based on a Disappearing-track Signature at √s = 13 TeV”
  - Tatsumi Nitta (Waseda University),“Search for the Weak Vector Boson Scattering in Semileptonic Final States in pp Collisions at √s = 13 TeV with the ATLAS Detector”
3. Theoretical nuclear physics:
  - Yuji Hirono (Asia Pacific Center for Theoretical Physics), “Topological Order in Gauge Theories of Gapless Superfluidity and its Appearance Condition”
  - Yuto Mori (Department of Physics, Faculty of Science, Kyoto University), “Toward Solving the Sign Problem with the Path Optimization Method”
  - Yasuhiro Yamaguchi (Japan Atomic Energy Agency), “The Mass Spectrum of Pc PentaQuarks in Hadron Dynamics”
4. Experimental nuclear physics:
  - Daiki Sekihata (Center for Nuclear Study, Graduate School of Science, University of Tokyo), “Measurement of Neutral Mesons and Direct Photons in pp and Pb-Pb Collisions at √s_NN_=5.02 TeV”
  - Takahiko Masuda (Research Institute for Interdisciplinary Science, Okayama University), “X-ray Pumping of 229Th Nuclear Clock Isomer”
  - Taiki Tanaka (Department of Nuclear Physics, Research School of Physics and Engineering, Australian National University), “Study of Quasielastic Barrier Distributions Towards Superheavy Nuclei Synthesis”
5. Cosmic ray and astrophysics:
  - Yutaro Enomoto (Department of Applied Phyics, School of Engineering The University of Tokyo), “Study of Interferometer Locking Scheme for Gravitational-wave Detectors”
  - Shigeo S. Kimura (The Frontier Research Institute for Interdisciplinary Sciences, Tohoku University), “Theoretical Study of High Energy Phenomena in Black Hole Accretion Flows”
  - Yuuki Wada (RIKEN Hakubi Research Teams, Extreme Natural Phenomena RIKEN Hakubi Research Team), “Studies of Photonuclear Reactions Triggered by Lightning Discharges”
6. Beam physics:
  - Takayuki Kubo (High Energy Accelerator Research Organization), “Theoretical Study on Super Conducting Accelerating Cavities”
  - Heishun Zen (Kyoto Univeristy Institute of Advanced Energy), “Research and Development of Improving performance of a Midinfrared Free Electron Laser”
7. Atomic and molecular physics, quantum electronics, radiation:
  - Tomoki Ozawa (Advanced Institute for Materials Research at Tohoku University), “Study of Geometric Effects, Especially of Quantum Metric, in Ultracold Atomic Gases and Other Synthetic Quantum Systems”
  - Fumihiro Kaneda (Frontier Research Institute of Interdisciplinary Sciences, Tohoku University), “Study of High-efficiency Single Photon Sources, Entanglement and Uncertainty Relations”
  - Shingo Kono (Center for Emergent Matter Science, RIKEN), “Quantum Control and Measurement of Itinerant Microwave Photons Using Superconducting Quantum Circuits”
8. Plasma:
  - Naoki Sato (Department of Complexity Science and Engineering, Graduate School of Frontier Sciences, The University of Tokyo), “Statistical Mechanics with Topological Constraints: Self-Organization in Foliated Phase Space”
  - Seiya Nishimura (Department of Electric and Electronic Engineering, Hosei University), “Theoretical Study on Kinetic Effects on Magnetohydrodynamic Instability”
9. Magnetism:
  - Hiroaki Ishizuka (Department of Physics, Tokyo Institute of Technology), “Theory of Asymmetric Scattering and Magnetic Transport by Magnetic Fluctuations”
  - Katsuhisa Taguchi (SEMITEC), “Theoretical Study of Inverse Faraday Effect and its Application”
10. Semiconductors, mesoscopic systems, and quantum transport:
  - Kohei Kawabata (Department of Physics, University of Tokyo), “Symmetry and Topology in Non-Hermitian Physics”
  - Ryutaro Yoshimi (RIKEN Center for Emergent Matter Science), “Experimental Study on Quantum Transport Phenomena in Topological Insulator Thin Films”
11. Optical properties of condensed matter:
  - Yohei Kawakami (Department of Physics, Tohoku University), “Photoinduced Phase Transitions and Strong Field Effects in Correlated Materials”
  - Hirokazu Tahara (Institute for Chemical Research, Kyoto University), “Quantum Coherent Dynamics of Photoexcited States in Semiconductor Nanostructures”
12. Metal physics (liquid metals, quasicrystals, low-temperature physics, ultralow temperatures, superconductivity, and density waves):
  - Satoshi Yui (Research and Education Center for Natural Sciences, Keio University), “Coupled Dynamics of the Two-Fluid Model in Superfluid ^4^He”
  - Yasuhiro Tada (The Univesrsity of Tokyo, Institute of Solid State Physics), “Theoretical Investigation on the Edge Current and Orbital Angular Momentum in Chiral Superfluids”.
13. Molecular solids:
  - Hiroshi Oike (Department of Applied Physics, The University of Tokyo), “Development and Control of Novel Electronic Phases in Organic Strongly Correlated Electron Systems”
  - Ryosuke Akashi (Department of Physics, The University of Tokyo), “First-principles Study on the Superconducting Phase in Hydrogen Sulfide Under High Pressure”
14. Strongly correlated electron systems:
  - Atsuo Shitade (Theoretical and Computational Molecular Science, Institute for Molecular Science), “Theoretical Study on Cross-correlated Responses in Crystals based on Electronic Multipole”
  - Shintaro Hoshino (Graduate School of Science and Engineering, Saitama University), “Theoretical Study on Electron Order in Strongly Correlated Electron Systems with Multi-orbital Degrees of Freedom”
  - Yuta Mizukami (Department of Advanced Materials Science, University of Tokyo), “Experimental Study on Electron Pairing Formation in Strongly Correlated Superconductors”
  - Yoshikazu Mizuguchi (Department of Physics, Graduate School of Science, Tokyo Metropolitan University), “Discovery of BiS_2_-based Layered Superconductors and Elucidation of Condition for Superconductivity”
15. Surfaces and interfaces, crystal growth:
  - Akitoshi Shiotari (Department of Advanced Materials Science, The University of Tokyo), “Control of Adsorption Structures and Valence States of Nitric Oxide at the Single-molecule Level”
  - Kuniyuki Miwa (Department of Chemistry, Northwestern University), “Theoretical Studies on the Optical and Transport Properties of Molecular Systems at the Nanoscale”
16. Dielectrics, ferroelectricity, lattice defects and nanostructures, phononic properties, and X-ray and particle beams:
  - Norihiro Oshime (National Institutes for Quantum and Radiological Science and Technology, Quantum Beam Science Research Directorate, Kansai Photon Science Institute, Synchrotron Radiation Research Center), “Ferroelectric Skewed Electronic Band Structure Induced by Electric Polarization”
  - Hikaru Saito (Institute for Materials Chemistry and Engineering, Kyushu University), “Electron Beam Spectroscopy for Plasmonic Crystals”
17. Fundamental theory of condensed matter physics, statistical mechanics, fluid dynamics, applied mathematics, socio- and econophysics:
  - Ryo Tamura (National Institute for Materials Science, International Center for Materials Nanoarchitectonics, MANA), “Development of a Method for Estimating Model Parameters from Experimental Data by Means of Machine Learning”
  - Tetsuhiro Hatakeyama (The University of Tokyo, Graduate School of Arts and Sciences), “Theoretical Study on Peculiar Dynamical Properties of Circadian Clocks”
  - Ryusuke Hamazaki (Nonequilibrium Quantum Statistical Mechanics RIKEN Hakubi Research Team), “Studies on Thermalization Processes of Isolated and Open Quantum Many-body Systems”
18. Soft matter physics, chemical physics, biophysics:
  - Tetsuya Hiraiwa (Mechanobiology Institute, National University of Singapore), “Mechanics of Cytoskeleton and Dynamics of Multi-cellular Tissues”
  - Fujihashi Yuta (Institute for Molecular Science, National Institutes of Natural Sciences), “Theoretical Study on Dynamic Processes in Complex Molecular Systems based on Quantum Science and Technology”
  - John Jairo Molina (Department of Chemical Engineering, Kyoto University), “Novel Soft Matter Physics through Computational and Information Science”

## Report on the 44th AAPPS Video Council Meeting Part I, December 1, 2020

The 44th Council Meeting of the Association of Asia Pacific Physical Societies (AAPPS) was held online from 10:00 a.m. to 12:30 p.m. KST (Korea Standard Time) on December 1, 2020, using a Zoom session prepared by the Asia Pacific Center for Theoretical Physics (APCTP). The participants were Jun’ichi Yokoyama (President), Hyoung Joon Choi (Vice-president), Nobuko Naka (Secretary), and council members Xiu-dong Sun (the Chinese Physical Society (CPS)), Ruiqin Zhang (the Physical Society of Hong Kong), Mio Murao (the Physical Society of Japan (JPS)), Akira Yamada (the Japan Society of Applied Physics (JSAP)), Woo-Sung Jung (the Korean Physical Society (KPS), APCTP), Kurunathan Ratnavelu (Malaysian Institute of Physics), Rajadeep Singh Rawat (Institute of Physics Singapore), Fu-Jen Kao (the Physical Society located in Taipei), and Meng-Fan Luo (the Physical Society located in Taipei). The meeting was observed by Baonian Wan (the Division of Plasma Physics (DPP)), Sang Pyo Kim (the Division of Astrophysics, Cosmology, and Gravitation (DACG)), Weiping Liu (the Division of Nuclear Physics (DNP)), Hiroyuki Nojiri (JPS), and Eunjeong Lee (AAPPS Liaison Officer). Keun-Young Kim (Treasurer), Gui-Lu Long (Ex Officio Member as Former President), and council members Jodie Bradby (Australian Institute of Physics), Tao Xiang (CPS), and Nguyen Quang Liem (Vietnam Physical Society) were absent.

(1) Secretary Naka reported the presence of 12 council members out of 17 members, and the quorum was met.

(2) President Yokoyama opened the 44th Council Meeting and welcomed all the participants, including division chairs Baonian Wan, Weiping Liu, chair-elect Sang Pyo Kim, and Hiroyuki Nojiri, who is a representative of the to-be-formed Division of Condensed Matter (DCM). Yokoyama mentioned that this council meeting was planned to be held in person in Hong Kong, but the meeting in person is postponed to next year.

(3) The agenda was adopted as prepared by the president.

(4) The secretary explained that the draft of minutes of the 43rd Council Meeting was circulated in August to the council members, and it was already published as a Society News article in the October issue of AAPPS Bulletin (AB). The president briefly explained the contents. The minutes were officially approved.

(4-1) Wan, the chair of DPP, reported on the current status and activities this year, on behalf of the board of directors. He reported that the articles of incorporation were revised by redefining the representative director as the chief executive officer. At DPP’s 3rd annual conference, which was held in Hefei, China, in November 2019, there were nearly 400 participants, which was a smaller number of participants than in previous conferences, due to the conference coinciding with the 14th Asia Pacific Physics Conference (APPC14). The 4th annual conference had originally planned to take place in Jeju, Korea, but ultimately, the conference was held as an e-conference. Special care was taken to assure the success of the conference online, e.g., by considering the time zone differences, assigning additional chairs to support each session, and preparing e-conference manuals and answers to frequently asked questions. There were 850 participants and 431 presentations, and the conference was very successful. Several prizes were awarded, and 12 articles were published in the Review of Modern Plasma Physics in 2020. Wan also introduced the 2021 fiscal year plans, which include preparations for DPP2021 in Fukuoka, Japan.

Liu congratulated the DPP for their activities, which were successful despite the Covid-19 pandemic. Wan added that the next conference is being planned for November 2021, with the hope that the participants will be able to meet in person.

(4-2) S.P. Kim presented activities of DACG on behalf of the division chair, Misao Sasaki. At APPC14, there was a joint session of DACG and Cosmology and Particle Astrophysics (CosPA). Several schools and workshops were endorsed by DACG in 2020. Approximately 70 participants attended the Asia Pacific School and Workshop held in Daejeon, Korea, in February 2020, with a slightly reduced number of participants due to the Covid-19 outbreak. In November 2020, the AAPPS-DACG workshop, supported by APCTP, was held online and attended by 213 participants. The best presentation awards were given to seven postdocs and students. CosPA2020 was postponed to November 2021 and will be held in Hong Kong. The DACG Executive Committee (EXCO) online meeting was held to elect the chair and to replace half of the EXCO members for the next term. S.P. Kim proposed an establishment of DACG awards aiming for APPC15, which will be discussed online.

(4-3) Liu, the chair of the Asian Nuclear Physics Association (ANPhA), presented the routine activities of the DNP. The annual board meeting will be held virtually in December 2020 and will be hosted in Hong Kong. ANPhA supported several meetings and young and talented researchers. In particular, he mentioned Dr. Nobuyuki Kobayashi, who won the CN Yang Award, and Dr. Bing Guo, ANPhA’s secretary, who was awarded the Youxun Wu prize from the CPS. An article reviewing the past 10 years of activities of ANPhA, edited by Prof. Tanaka, was already published, and the organization of the nuclear astrophysics progress report in Asia will be finished soon. Liu participated in the Nuclear Physics European Collaboration Committee as an observer. Two nuclear physics programs have started the A3 Foresight Program to establish a top-level and in-depth research hub in Asia. Liu expressed many thanks for the support and guidance of AAPPS.

S.P. Kim requested to share the presentation slides with other divisions and Liu agreed. Yokoyama summarized that he is pleased to know that all three divisions are actively functioning and promoting physics in the Asia Pacific region.

(5) Nojiri reported on the proposal of the formation of the DCM. He explained that the discussions regarding the formation of the DCM started approximately 2 years ago. At the beginning of 2020, work divisions and a timeline were prepared, but the process was slowed down because of the Covid-19 pandemic. The bylaws were distributed in late November 2020. The documents will be finalized in a meeting of representatives from five societies (CPS, Indian Physics Association, KPS, the Physical Society located in Taipei, JPS) and one observer (JSAP). They will join the round table on December 5, 2020, to found the DCM. If approved, a call will be made for other societies that have not been included so far. Nojiri explained that the division aims to promote the exchange and sharing of scientific knowledge and scholars. Membership can be given to regular and associate members, including students approved by an EXCO member. The core areas are diverse, and thus, boundaries of the division are difficult to define.

Yokoyama asked about the status of JSAP, and Akira Yamada answered that Prof. Tanaka will give some information in the coming meeting as the delegate from JSAP. S.P. Kim asked how one can become a member of the DCM. So far, each division is incorporated within each society, and each member should have an affiliation to some of the member societies of AAPPS. Meanwhile, nonmembers of each society also could apply to receive membership into each division upon recommendation by an EXCO member. S.P. Kim suggested that each division should set up a guideline for such a procedure. Nojiri answered that the DCM will ask for individual registration upon application. Later, it could be shifted to group registration when the number of members is increased.

Hyoung Joon Choi commented that the participation of as many individual members as possible is important for successful division formation. He suggested to call for inaugural members when the division is ready to be officially established. Nojiri responded that they can call for the participation of as many different societies as possible in the meetings before the formal start on January 1, 2021.

(6) Kurunathan Ratnavelu reported on the proposal for the establishment of the Division of Computational Physics. Since 2012, AAPPS has created divisions to reflect the growing stature of physics in the Asia Pacific region. He explained that the Division of Computational Physics (DCOMP) in the American Physical Society (APS) was founded in 1986, and the Computational Physics Group in the European Physical Society (EPS) was established much earlier in 1972. As is seen in the focus topics lead by DCOMP for the APS March Meeting 2021, computational physics cut across all branches of physics. Organizing the Workshop for Computational Physics, to be held in November 2021 in Kuching, Malaysia, will be a good place to start forming a division or a topical group in AAPPS.

Ruiqin Zhang commented that a more detailed proposal will come next year. Some of the audience at the International Union of Pure and Applied Physics (IUPAP) conference, organized by Zhang in Hong Kong, can be invited to the next meeting. Yokoyama asked if any society already has a division of computational physics. Ratnavelu answered that they have a group for theoretical and computational physics in Malaysia. Zhang mentioned that in China, there is a journal. Liu informed that there is a subsociety of computational physics under CPS. Yokoyama explained that JPS does not have a division of computational physics, but there are some groups of people who are trying to establish one. Yamada stated that many presentations related to computational physics are given in the biannual domestic conferences of JSAP, but there is no unified division. Ratnavelu asked about the status of the proposal for the quantum information division. Yokoyama answered that it has been suspended. Yokoyama showed interest to formulate topical or cross-disciplinary groups in AAPPS, as in APS and EPS. S.P. Kim commented in chat that AAPPS bylaws allow regular or interdisciplinary divisions (http://aappsbulletin.org/myboard/read.php?Board=others&id=24).

(7) Ratnavelu reported on the current status of the Malaysian Institute of Physics. There are 1506 members and 17 council members holding seven meetings per year. Their journal is indexed in the Emerging Science Citation Index. There are two divisions: medical physics and plasma physics. The Medical Physics Division supported travel grants and conferences. The Malaysian Institute of Physics hosted APPC14 and sent two students to the Asian Oceanian Forum with travel grants. The proceedings of APPC14 are to be published in January 2021.

(8) Woo-Sung Jung reported on the ongoing cooperation between APCTP and AAPPS. The APCTP was established in 1996 on the campus of Pohang University of Science and Technology and approved as the administrative headquarters of AAPPS in 2016. APCTP financially supports AAPPS through various aspects, in areas such as the Council Meetings, APPC, AB publication, and division activities. The support to each division started in 2017, and the staff members work as administrative editors of AB. The CN Yang Awards, which are currently disbursed in partnership between AAPPS and APCTP, were changed and became annual awards. The timeline for the selection process for next year was presented.

(9) Jung reported on the current status of AB on behalf of Gui-Lu Long, the editor-in-chief of AB. The annual editorial meeting this year was held online, in addition to the monthly video meetings. Five member societies (CPS, JPS, JSAP, KPS, the Physical Society located in Taipei) and APCTP financially contributed to the printing and editorial meetings. He thanked the cooperating members who were invited to be authors. In order to publish more scientific papers, the editorial board has been reorganized. As deputy editors-in-chief, Jung takes charge of administrative issues regarding the partnership with Springer Nature while YB Sheng is in charge of scientific issues. From January 2021, AB will be published by Springer Nature. The plan, set by the editorial board, is to publish 70 articles in 2025 and to promote the journal through Springer Nature’s channels, with indexing in the future.

S.P. Kim commented that the editorial board should announce the scope of the articles. Jung answered that in terms of disciplines, the AB covers every area of physics, and in terms of paper categories, most of the articles to be published next year will be review articles. In 2022, the hope is that 10–20% of the articles will be regular articles. S.P. Kim highly appreciated Jung’s efforts and mentioned his optimistic view that some authors may submit their original research papers, once every article in AB is indexed, not necessarily by SCOPUS or SCIE but by Springer Nature. Jung answered that indexing in SCOPUS in 2 years (2023) and SCIE in another 2 years (2025) is planned.

Jung reported that the contract with Springer Nature was signed in October 2020. Yokoyama asked Jung who signed the contract with astonishment. Jung answered he himself signed it as a council member of AAPPS to make quick progress after consulting with the editor-in-chief, Prof. Gui-Lu Long. It should have been consulted to the council and to the president beforehand, and Yokoyama voiced that AAPPS cannot give any financial commitment. Jung admitted his mistake and agreed that APCTP deals with Article Processing Charge (APC). S.P. Kim suggested that for the future the contract should be signed at the “top level,” i.e., between AAPPS and Springer Nature presidents. Jung will propose this to Springer Nature.

Yokoyama pointed out that from the authors’ side, the template prepared by Microsoft Word is not comfortable for writing science-oriented reports. Jung explained that Springer Nature will provide a LaTex template from January 2021, and the new online system could accept both Microsoft Word and LaTex formats. Yokoyama expressed his thanks to APCTP not only for financial but also administrative support, and particularly to Ms. Eunjeong Lee for her dedicated help.

(10) Yokoyama and Choi recently learned that IUPAP does not have any permanent legal personality in any country, but they take a legal personality or some appropriate status every 6 years when their administration office is moved. As the AAPPS administration office is in APCTP, the possibility of taking legal personality according to the law of Korea has been researched by Choi and Jung.

Choi explained as follows. In Korea, a bank account can be given only to a legal entity, i.e., to a real person or a person in law. Although the bank account of AAPPS is presently in the treasurer’s name, the Korean government recognizes that the assets belong to AAPPS rather than to the treasurer. To have a legal entity, a Board of Trustees (BT) consisting of nine or fewer trustees and one auditor is suggested. The legal entity should be independent and should make its own decisions on financial and accounting issues.

Choi explained that to abide by the law, full members of the BT should come to Pohang to establish physical presence every year. So, it is not convenient practically for full council members to participate in the BT. S.P. Kim pointed out that it is important to maintain a BT in harmony with the AAPPS Council. Choi responded that everything works based on trust. More study is required on these issues.

(11) Choi reminded the council meeting participants that 21 months are left before APPC15, which will be held in Gyeongju, Korea, starting on August 22, 2022. The floor plan and a tentative timeline were introduced: the first announcement (June 2021), the list of invited speakers (January 2022), the submission deadline (April 2022), notification of acceptance (May 2022), and the final schedule (June 2022). A council meeting has been planned to take place on August 21, 2022. The topics for APPC15 will include all fields of physics. We wish to request the participation of all (probably five) divisions and division chairs to take charge of sessions in their areas of expertise.

Other issues to be decided are organizing committees, participation of other organizations in the Asia Pacific region, publication of proceedings, and financial support. S.P. Kim expressed concern that inviting speakers 7 months before the conference might be too late. Choi answered that the date is the last moment to finalize the list. All contact needs to be made early; as many events were canceled in 2020/2021, 2022 may be a particularly busy year with many events. Liu agreed to this point. Yokoyama suggested to have a dedicated council meeting with the division chairs in March.

S.P. Kim asked how many participants are expected. Choi answered that the first goal is more than 1000, and it will be very successful if it is more than 2000. Wan suggested the possibility to combine APPC15 with annual division meetings. Liu agreed that if many divisions come in conjunction with this event, it could encourage interdisciplinary intersections with different divisions. Ratnavelu asked how long the conference will be, and Choi answered that he anticipates 4 days, from Monday to Thursday. S.P. Kim added that Gyeongju is a wonderful and convenient place for conferences.

(12) President Yokoyama announced that the meeting will be continued to the second part, scheduled on December 15, 2020, and closed the first part of the meeting.

## Report on the 44th AAPPS Video Council Meeting Part II, December 15, 2020

Following the first part of the 44th Council Meeting of the Association of Asia Pacific Physical Societies (AAPPS) on December 1, 2020, the second part of the 44th Council Meeting was held online from 10:00 a.m. to 12:25 p.m. KST (Korea Standard Time) on December 15, 2020, via Zoom session hosted by the Asia Pacific Center for Theoretical Physics (APCTP). The participants were Jun'ichi Yokoyama (President), Hyoung Joon Choi (Vice-president), Nobuko Naka (Secretary), Keun-Young Kim (Treasurer), Gui-Lu Long (Ex Officio Member as Former President), and council members Jodie Bradby (Australian Institute of Physics (AIP)), Xiu-dong Sun (the Chinese Physical Society (CPS)), Tao Xiang (CPS), Mio Murao (the Physical Society of Japan (JPS)), Akira Yamada (the Japan Society of Applied Physics (JSAP)), Kurunathan Ratnavelu (Malaysian Institute of Physics), Rajadeep Singh Rawat (Institute of Physics Singapore), Fu-Jen Kao (the Physical Society located in Taipei), and Meng-Fan Luo (the Physical Society located in Taipei). The meeting was observed by Youngah Park (the Korean Physical Society (KPS)), Sang Pyo Kim (the Division of Astrophysics, Cosmology, and Gravitation (DACG)), Hiroyuki Nojiri (JPS), and Eunjeong Lee (AAPPS Liaison Officer). Council members Ruiqin Zhang (the Physical Society of Hong Kong), Woo-Sung Jung (KPS, APCTP), and Nguyen Quang Liem (Vietnam Physical Society) were absent.

(1) Secretary Naka reported the presence of 12 council members out of 17 members, and the quorum was fulfilled. [Note added: number of council members present during the meeting changed from 12 to 14].

(2) Agenda of the second part of the 44th Council Meeting was adopted as prepared by the president.

(3) Youngah Park, the chair of the Women-in-Physics Working Group of AAPPS (AAPPS-WIP) reported on recent activities. She first introduced the history of AAPPS-WIP and its current members. The working group was established after the approval of the 16th AAPPS council meeting held in Osaka, Japan, in April 2006. The first workshop on AAPPS-WIP was held at APPC10 in 2007, and the most recent workshop was held at APPC14 in Sarawak, Malaysia, in November, 2019. Seven talks were delivered in the AAPPS-WIP session of APPC14, and extended working group members had a luncheon meeting after the session. The working group recently made a AAPPS-WIP homepage for networking. Park acknowledged support from AAPPS and APCTP. Park explained that the International Conference on Women in Physics (ICWIP2020), which had originally been planned to take place in Melbourne, Australia, in July 2020, was postponed to July 2021 due to the Covid-19 pandemic. The conference will be held virtually, as it will take some time to make the new vaccines globally available. The best and easiest platform to be used is under exploration. The IOP Journal of Physics: D is planning to publish 2021 Women in Applied Physics Roadmap. From 18 to 20 pieces of short reviews on every aspect of applied physics will be contributed by all the female authors.

Furthermore, Park introduced the activities of Gender Equality Promotion Committee in JPS. The activities were initiated and conducted based on large-scale survey for the last twenty years by the Japan Inter-Society Liaison Association Committee for Promoting Equal Participation of Men and Women in Science and Engineering. A new award for young female scientists after the name of Prof. Fumiko Yonezawa was recently established in JPS. Summer camps for high-school girls, which were held for 15 years since 2005, are very successful. The camp was held online this year. The committee has been organizing symposiums and luncheon meetings in annual JPS meetings every year since 2002. They also provide daycare services for participants’ children in JPS meetings since 2010.

Park also reported on the activities of the Women in Physics Committee in Korea. The number of women is approximately 20 % of the total number of the KPS members. The committee was established in the year 2002 in Busan, Korea. Since 2002, the committee organizes physics camps for high-school girls, site visits, and special sessions in KPS meetings. The surveys were conducted in 2005, 2010, and 2014, and the female ratio is found to be continuously improving. Nevertheless, there are still concerns, such as only 20 % of the female scholars have children and women feel more pressure for parenting than men. The standing of Korea in the index of gender inequality is 17th out of 152 countries. Yokoyama suggested to share the presentation slide to which Park agreed.

(4) Hiroyuki Nojiri explained the preparation status for the formation of the Division of Condensed Matter Physics (DCMP) as a regular division. A preparation for the foundation and a round table were held on December 4 and 5, 2020. He summarized the purpose, core areas, and core starting members for the division. The division will form an executive committee (EXCO), consisting of two representatives from each of the five founding societies and one or two representatives from each of the newly participating societies depending upon size. The division sets the status of an observer (not participating but having interest to receive information) by the bylaws. Prof. Je-Geum Park is the candidate division chair and Nojiri will be a vice chair. The selection of another vice chair and auditors are under progress. The membership will be given to regular members of Ph.Ds. or equivalent degree holders as well as to associate members including students. Registration will be completed on personal basis; Anyone belonging to the participating societies of AAPPS can join. The division will also accept exceptional cases, such as a researcher who lives in an area where no AAPPS society exists. The division defines the codes of conduct. Article 6 declares gender equality in composition and activities. Nojiri explained the schedule toward the foundation. The bylaws have been circulated for the formal signing by 30 founding members from the five societies. Finalization of the bylaws and nomination of the second vice chair will be completed by December 20, 2020. The founding documentation will be further circulated to call for participation to the division. The web site is presently under preparation and will be complete in March 2021. The first division meeting is planned to be held in November 2021 in Korea as a hybrid conference. Yokoyama stated that he was impressed by the sophisticated bylaws of DCMP. S.P. Kim commented that minimized simple bylaws are better, and to let the division work functionally is important. Yokoyama responded that respective divisions go its own way and amendment can be made at any time whenever it becomes necessary.

Tao Xiang asked whether the list includes full members. Nojiri answered that it is only a part of the list of 30 founding members. Choi asked how one can join the division. Nojiri responded that the division not only asked five founding societies to nominate six representatives, but also will call for initial members other than the representatives. S.P. Kim asked how EXCO members are nominated from each society. He suggested steps along the line of AAPPS philosophy, such as recommendation from the president of each society for an EXCO member. Akira Yamada asked about the membership fee, and Nojiri answered that membership is free. Nojiri expects that the division does not need a large financial source at the beginning and handling assets is too complicated at the time of foundation. Jodie Bradby mentioned that there is no separate condensed matter group in Australia and New Zealand but is part of the AIP groupings. Yokoyama asked Bradby to announce the foundation of DCMP to Australian community and to encourage them to join. Rajadeep Singh Rawat stated that in Singapore, the condensed matter society is not very large while the material science community is rich and huge. He asked if DCMP has a plan to establish a legal entity status. Nojiri answered the division plans for transfer to this status in six years, and the transfer is intended only as a technical measure to treat financial issues. S.P. Kim added that even after DPP was legalized, the division is not independent of AAPPS and is called AAPPS-DPP.

S.P. Kim proposed to build up a systematic divisional website at the AAPPS headquarter, i.e., at APCTP, so that one could see contents of all divisions at once. Yokoyama stated that all division websites are already linked from the AAPPS main page. Nojiri explained that the domain name of DCMP has not yet registered. Yokoyama stated that it is possible to have more than two domain names for a single website, and suggested Nojiri to specify the second name under the AAPPS domain name from a systematic point of view for the readership. Fu-Jen Kao voiced that JSAP is the largest society and seems to run one of the most successful condensed-matter related conferences in the Asia Pacific region. Therefore, cohosting a member-society’s annual conference with the division meeting seems to be very efficient and interesting. S.P. Kim also hoped JSAP to join DCMP, as JSAP and JPS are two independent member societies of AAPPS. Yokoyama summarized that active participation from JSAP is strongly encouraged.

Meng-Fan Luo stated that although the Physical Society located in Taipei does not have many participating members at present, more members will attend in future. In Taiwan, they have recently established a new division of particles and fields, and biophysics and computational physics divisions will be established in coming future. The condensed matter group covers a large area, and it is separated into several smaller divisions.

Xiang stated that six core members participating to DCMP are from CPS. Each division of CPS, associated with condensed matter, has a representative. Xiang explained that CPS organizes annual meetings of condensed matter in July each year. There is a possibility to combine this with the division meeting as a joint meeting.

(5) President Yokoyama proposed a motion to vote for the approval of the establishment of DCMP in AAPPS. The motion was unanimously agreed. Yokoyama congratulated Nojiri. For the next formal process, approval from not less than four member societies is required according to the bylaw.

(6) Treasurer Kim reported the financial status of AAPPS. He explained that in 2019 we started with a carry-over of KRW 52,000,000 and the balance as of December 15, 2020 was KRW 63,000,000 (USD 53,000) which is a surplus. The Leo Koguan Foundation has USD 36,500 after USD 10,000 has been excluded for AAPPS Bulletin (AB). The major income comes from AAPPS’s membership fees, which are USD 500 for each society. The major expenses are the certificate case and shipping costs for AAPPS activities. Yokoyama expressed concern that the number of societies paying the membership fees is continuously decreasing. Bradby mentioned that the invoice may be missed at the time of transition to the new staff members [*]. Five societies are donating USD 5,000 each as AB contribution. The support from APCTP is USD 358,050 since 2004, and DACG covers the “aapps org” domain fee for the next five years. [*Note added: this was subsequently resolved by the treasurers and the AIP did actually pay their invoice].

(7) Kao, the chair of the CN Yang Award selection committee reported on the current situation and future perspective of the CN Yang Award. He explained about the timeline of the 2020 Awards and the selection process of the awardees. The call started on February 3, 2020, and nominations were closed on April 15. After initial screening to check the eligibility, the selection committee was formed. Reviewers were appointed by the committee and the evaluation reports were prepared by the reviewers approximately till the end of May. The exchange of opinions and online meetings were started five weeks later so that the committee members could have enough time to think carefully. Each winner delivered a talk at the award ceremony held on November 5, 2020. Among 23 nominees in nine categories, those in three categories covered by the AAPPS divisions were pre-screened by each division while others were evaluated by reviewers. The committee received written evaluation reports and selected 11 candidates (maximum three from each category), based on the scrutinization of the reports and discussion. Afterwards, five semifinalists were chosen by voting and finally three winners were chosen by voting. The voting was conducted by committee members excluding the chair.

There are some feedbacks from the last-year meetings. The main issues are i) lack of basic statistics regarding a candidate as a reference, ii) insufficient criticalness in review report to qualify the candidate, and iii) many do not know that an individual can nominate. Kao proposed minor amendments in the evaluation form.

In the current rule, the selection committee consists of members chosen from three categories, i.e., AAPPS council, AAPPS divisions and APCTP science council, and the number of members from each category is set to be equal. The number of divisions will soon become five. The increase in number # may lead to expansion in the size of the committee, thus may cause difficulty in coordination. Therefore, Kao proposed to limit the number of the committee members up to 12 provided that the chair is not counted in. In the maximum case, (8-#), #, and four committee members will be allocated to AAPPS council, AAPPS divisions, and APCTP science council, respectively. S.P. Kim asked how to strike a balance between societies. Kao answered that the role of each society is to nominate, and selection is made by the committee.

Gui-Lu Long proposed routine of the subjects in the CN Yang Awards, in order to protect some minority subjects from disciplines having many recipients. Kao responded that the committee selects the best candidates each year and the relative impact and standing of nominees in his/her category really count. Yokoyama mentioned that we should try to cover all the fields of physics as much as possible in a long run. Long suggested to set subjects of the two recipients rotating and the remaining one free, among three recipients. Kao said this would be a very good consideration and will become a consensus rather than a written rule. Long agreed to this point.

Subsequently, Kao voiced that considering the foundation of new division(s), in particular the huge areas covered by DCMP, we shall relax the rule for pre-screening to allow maximum three nominees from each division rather than one in the current rule. This opinion was seconded by Luo and S.P. Kim. Yokoyama once again clarified the current process: We call for nominations to member societies and participants of APPC. For nominees those who have been classified to corresponding divisions, their application forms are sent to the division chair. The division chair forms a sort of sub-selection committee inside the division for pre-screening, afterwards, one candidate is chosen from the division. The number of pre-screened candidate(s) might be changed from one to three, considering the new division formation.

Kurunathan Ratnavelu asked whether the number of committee members will be 12 or remain 9 next year. Yokoyama responded that it will be 12 if the current rules are applied and the new division, DCMP, is included. Finally, Kao proposed to limit the maximum number of selection committee members up to 12. The motion was unanimously agreed. Yokoyama added that we need to consult with APCTP as well. Written amendments of rules will be circulated by the next council meeting and the rules should be fixed by April, 2021.

(8) Keun-Young Kim reported on the progress of the establishment of the Division of Particles and Fields (DPF). The first discussion started after the banquet of APPC14 in 2019. He introduced the list of representatives from five societies, i.e., AIP, CPS, JPS, KPS, and the Physical Society located in Taipei. After two meetings, a small working group was formed. The first working group meeting will take place soon, after Christmas or in the new year. Yokoyama asked about the possibility of participation from India.

(9) In 2017 under the leadership of the former president Long, AAPPS determined the rules on the sponsorship, co-sponsorship, and endorsement of AAPPS activities. The only case applied to the sponsorship or co-sponsorship so far is APPC without any problem. Yokoyama proposed to amend the application due date for endorsement, which is four months in advance in the original rule. As approval in email communications does not need a long time, the proposed deadline is three weeks before the desired notification date. The amendment was approved by the council members. Yokoyama introduced Article 3 on the criteria, which must be satisfied to be sponsored, co-sponsored, or endorsed. DACG and DCMP incorporated these terms in their bylaws as codes of conduct. Yokoyama hopes other divisions to do so as well.

(10) President Yokoyama presented the list of his activities following the 43rd Council Meeting. He personally attended the JPS meeting held online, as the president, he attended the Meeting of the Physical Society of Philippines held online in October, the DPP annual meeting, CN Yang Award ceremony, the joint meeting of KPS and JPS in November, and DACG’s first online conference in November 2020. At the 43rd Council Meeting, he proposed to establish a joint award between AAPPS and each member society. He is collecting information from each society about the type of awards being provided, especially for young researchers. He is sometimes asked about merit of being an AAPPS member. Establishment of such an award may help to answer this question. He hopes to provide joint presentation awards at the 4th Meeting of the Physical Society located in Taipei as a pilot case. He calls for other societies if interested in such a joint award.

(11) President Yokoyama summarized the last meeting, i.e., 44th Council Meeting, Part I. There were reports from division chairs on division activities. Progresses toward the formation of DCMP and the Division of Computational Physics (DCP) were reported. Yokoyama attended the preparation meeting for the division of computational physics inside JPS, and introduced possible activities of AAPPS next year. Any progress on AB will be reported by email. Status of a legal entity is difficult to achieve in a short time. We will continue discussion on APPC15 and will have a council meeting in March, 2021. Yokoyama added that the forthcoming APPC will be a much larger conference than Choi presently anticipates, and suggested possibility of 4.5 or up to 5 days for the sessions.

(12) President Yokoyama announced that the next council meeting will be scheduled in March 2021, and closed the meeting.
